# Rapid regional mobile laboratory response and genomic monkeypox virus (MPXV) surveillance in seven East African Community partner states, August 2024: preparedness activities for the ongoing outbreak

**DOI:** 10.2807/1560-7917.ES.2024.29.35.2400541

**Published:** 2024-08-29

**Authors:** Florian Gehre, Eric Nzeyimana, Hakim Idris Lagu, Emmanuel Achol, Julien A Nguinkal, Eric Kezakarayagwa, Théogene Ihorimbere, Néhémie Nzoyikorera, Francine Kabatesi, Marie-Noelle Uwineza, Abdi Roba, Millicent Nyakio Ndia, John Ndemi Kiiru, Gwokpan Awin Nykwec, Isaac Gatkuoth Chot Moun, Mamdouh A Aguer, James A Maror, Gregory Wani Dumo, Michael Losuba, Lul Lojok Deng, Neema Omari, Grace Ochido, Aryse Martins Melo, Peter Bernard Mtesigwa Mkama, Edna Mgimba, Monica Fredrick Francis, Lawrence A Mapunda, Alex Magesa, Nyambura Moremi, Godfrey Pimundu, Tonny Muyigi, Susan Ndidde Nabadda, Emmanuel Kabalisa, Isabelle Mukagatare, Daniel Mukadi-Bamuleka, Erick Ntambwe Kamangu, Jürgen May, Muna Affara

**Affiliations:** 1 Department for Infectious Disease Epidemiology, Bernhard-Nocht-Institute for Tropical Medicine, Hamburg, Germany; 2 East African Community Secretariat (EAC), Arusha, Tanzania; 3 National Institute of Public Health, Ministry of Health and Fight Against AIDS, Bujumbura, Burundi; 4 National Public Health Laboratories, Ministry of Health, Nairobi, Kenya; 5 National Public Health Laboratory, Ministry of Health, Juba, South Sudan; 6 Ministry of Health, Dodoma, Tanzania; 7 National Public Health Laboratory, Dar es Salaam, Tanzania; 8 National Health Laboratory and Diagnostic Services (NHLDS), Ministry of Health, Kampala, Uganda; 9 Biomedical Services Department, Biomedical Centre Rwanda, Kigali, Rwanda; 10 National Institute for Biomedical Research, INRB, Ministry of Health, Prevention and Social Foresight, Democratic Republic of the Congo; 11 National Institute for Public Health (INSP), Kinshasa, Democratic Republic of the Congo; 12 German Center for Infection Research (DZIF), partner site Hamburg-Lübeck-Borstel-Riems, Hamburg, Germany; 13 Tropical Medicine II, University Medical Center Hamburg-Eppendorf (UKE), Hamburg, Germany

**Keywords:** Mpox clade Ib, EAC Mobile Laboratory, genomic surveillance, East African Community, Outbreak response

## Abstract

The East African Community (EAC) is experiencing an unprecedented, emerging mpox outbreak since July 2024 in five of eight partner states. We highlight rapid regional response measures, initiated August 2024 coordinated by EAC: field deployment of six mobile laboratories in Burundi, Rwanda, Uganda, Tanzania, Kenya, South Sudan to high-risk areas, donation of one mobile laboratory to Democratic Republic of the Congo and genomic monkeypox virus (MPXV) surveillance support. These interventions aim to limit local mpox spread and support international containment.

In 2022, a global, multi-country mpox outbreak was reported, and since 2024 the novel monkeypox virus (MPXV) clade Ib is expanding in countries of the East African Community (EAC). The EAC is a regional economic community and the intergovernmental body of eight partner states: Burundi, Democratic Republic of the Congo (DRC), Kenya, Rwanda, Tanzania, Somalia, South Sudan and Uganda [1]. In early 2024, the novel MPXV clade Ib was detected in eastern DRC and, in July, in four more EAC partner states, with the remaining countries at risk of spillover events. Here, we outline the steps the EAC is currently coordinating in seven of its eight partner states as part of its regional mpox response, genomic surveillance (for MPXV clade Ib identification) and preparedness activities.

## Outbreak detection

All World Health Organization (WHO) regions affected by the mpox oubreak in 2022 reported steadily declining case numbers by June 2024, with the exception of the WHO Africa region, which documented an increase of cases by 22%. This increase was mainly driven by a sharp rise of laboratory-confirmed mpox cases in the DRC, where 14,626 cases were notified in 2023 and 7,851 cases in 2024, up to 26 May [2]; the eastern part of the country was particularly affected (Figure).

**Figure:**
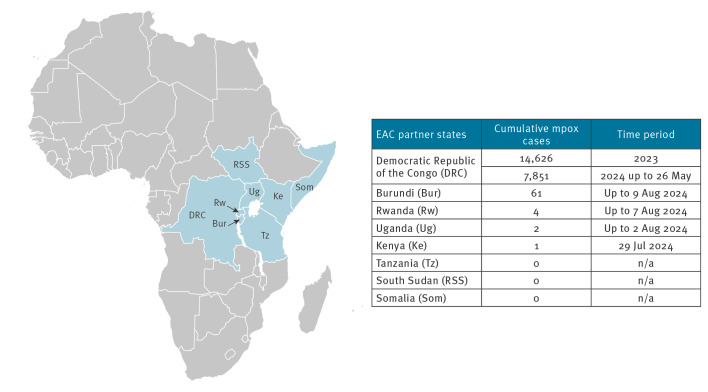
Distribution of mpox cases, East African Community, up to 12 August 2024 (n = 22,545)

EAC: East African Community; n/a: not applicable.

Source [2].

The emergence of a new MPXV variant clade Ib in the eastern DRC has possibly accelerated transmission, and extended the infection beyond DRC borders to the wider EAC region. Apart from the DRC, spillover events or new outbreaks of MPXV clade Ib were documented in Burundi (n = 61 cases), Rwanda (n = 4 cases), Uganda (n = 2 cases) and Kenya (n = 1 case) in 2024 up to 12 August [3]. South Sudan and Tanzania, which neighbour the affected countries, have not reported any mpox cases yet. With seven EAC Partner States directly affected by mpox or at-risk, the EAC region is currently a global hotspot. Consequently, on 13 and 14 August 2024, respectively, the Africa Centre for Disease Control (Africa CDC) and the WHO declared mpox as a ‘Public Health Emergency of continental security’ [4] and ‘Public Health Emergency of International Concern (PHEIC)’ [5].

## Establishment of the EAC mobile laboratory network

Since 2017, the EAC has established a network of nine modular mobile laboratories in the original six EAC partner states (Burundi, Kenya, Rwanda, Tanzania, South Sudan and Uganda) for the purpose of viral haemorrhagic fever diagnosis. This network is technically supported by the German Bernhard-Nocht-Institute for Tropical Medicine (BNITM), with funding from the German Federal Ministry for Economic Cooporation and Development (BMZ) through the German Development Bank (Kreditanstalt für Wiederaufbau, KfW). In 2020 the mobile laboratories were handed over to respective countries where they are operated by the National Public Health Laboratories (NPHLs)/Ministries of Health, in addition to their full integration into national outbreak response management. 

The mobile laboratories are classified as Type II (box-based) rapid response mobile laboratories (RRMLs), according to WHO [6]. Each laboratory consists of sample reception areas, mobile gloveboxes (‘Ultra Glove Box’, Koennecke, Berlin, Germany) for sample inactivation for Risk Group 3 and 4 pathogens, PCR hoods and BioRad CFX-96 Real-Time PCR machines, and, if needed ELISA modules for serology [7]. Type II mobile laboratories can be set up in existing infrastructures (such as peripheral health centers) at the site of the local outbreak (i) to substantially shorten the diagnostic turnaround time to results release, (ii) to avoid sample transportation from site of outbreak to the centralised NPHL (during which collected specimen can degrade) [7] or (iii) to circumvent other logistical challenges such as cost of sample shipment and power supplies for cold chain devices and storage units.

In the framework of the EAC mobile laboratory network, harmonised diagnostic standard operating procedures (SOPs) were developed and rolled out across the EAC by training over 72 mobile laboratory operators from six NPHLs in diagnosing arbo- and haemorrhagic fever viruses [7]. Most importantly, an extensive MPXV diagnostics training was also conducted in five EAC countries (Burundi, Kenya, South Sudan, Tanzania, Uganda) in 2022 [8].

## Rapid response activities to the mpox outbreak

Given the current geographical expansion of the mpox epidemic in the EAC, and in response to Africa CDC’s alert, the EAC Secretariat is activating the established resources in August 2024 in order to commence regional mpox mobile laboratory response and preparedness activities. The value of the mobile laboratory network has been previously demonstrated during the SARS-CoV-2 pandemic (2020–22) [9], Sudan virus disease outbreak (Uganda, 2022), Marburg virus disease outbreak (Tanzania, 2023), Yellow fever outbreak (South Sudan, 2024), and various WHO simulation exercises. This will enable timely response in the early phase of the epidemic at a national and/or regional level.

### Activation of the mobile laboratory network

The EAC will make seed funds available to enable NPHLs of the six original EAC partner states and mobile laboratory network members, to deploy their mobile laboratories to strategic high-risk areas. This initial funding will be used to support one mobile laboratory per country. These seed funds will therefore bridge the time gap until additional national or international financial resources can be mobilised, as they will cover six laboratory personnel, all logistical costs and costs of diagnostic FlexStar Monkeypox virus PCR Detection Mix 1.5 kits (Altona Diagnostics GmbH, Hamburg, Germany). Uganda has already deployed their mobile laboratory and Burundi is finalising logistics for immediate deployment.

As the DRC was not part of the original initiative (and only joined the EAC in 2022), yet is currently the most affected EAC partner state, the EAC Secretariat will donate one mobile laboratory to the country. The laboratory will reinforce MPXV screening at the Rodolphe Merieux Institut National de Recherche Biomédicale in Goma (North Kivu) to provide diagnostics to remote populations in North and South Kivu provinces, on the DRC’s eastern border. 

### Preliminary data collection from mobile laboratory intervention

In Uganda, one EAC mobile laboratory was deployed on 24 July 2024 to Bwera hospital, where it serves the Kasese district and four neighbouring districts, including the Mpondwe DRC–Uganda border post. The border has a high volume of people seeking healthcare, visiting markets and/or relatives or friends in Uganda, or transiting to other East African countries. Suspected mpox cases according to the WHO case definition [10] can be screened at the mobile laboratory with a turnaround time of 3 h (upon sample receipt). This is in contrast to the 24 h time period required for samples sent to the centralised laboratory in Entebbe. Up to 26 August 2024, 31 samples (n = 17 females and 14 males) were processed, and no positive cases were detected.

In Burundi, Kayanza province has a large number of suspected mpox cases. As no laboratory capacity for MPXV diagnostics has been previously available, samples must be transported to the NPHL in Bujumbura, which is logistically difficult, and given long transport times and therefore delayed diagnostics, the patient results are only available after 24 h. A mobile laboratory is currently being deployed to Kayanza, which will eliminate complicated, time-consuming sample transports and reduce the diagnostic turnaround time to an expected 3 h.

### Strengthening mpox genomic surveillance

An additional key intervention of the EAC response is to strengthen the mpox genomic surveillance activities in the region in order to monitor emergence of new variants and potential vaccine escape virus mutants, and to establish the molecular epidemiology and transmission dynamics of MPXV. For that purpose, the EAC is currently operationalising a regional MPXV sequencing network. The network will be one of Africa's largest Illumina-based sequencing infrastructures, equipped with 6 NextSeq1000 at central NPHLs and 12 iSeq100 in mobile laboratories. This capacity, coupled with an already trained cadre of 12 bioinformaticians and 12 wet laboratory experts (‘Trainers-of-Trainers’; ToTs) from the NPHLs, provides an important platform for mpox genomic surveillance. 

In conjunction with laboratory deployment, the EAC network will conduct an mpox specific refresher ‘wet lab’ and bioinformatics training in Burundi for the ToTs, which in turn will train further staff during ’in-country trainings’. Through the NPHLs, this genomic surveillance network will be linked to international initiatives and global surveillance efforts.

## Discussion

The spread of the MPXV clade Ib across the EAC region is also of relevance for countries outside of the African continent. The multi-country outbreak in 2022 – caused by MPXV clade 2 – spread globally, with 99,176 laboratory confirmed cases in 116 countries [3]. In the present outbreak, single cases outside of Africa suggestive of MPXV clade Ib infection were reported in Sweden [11] and Thailand [12]. According to an epidemiological update published by the European Centre for Disease Prevention and Control (ECDC) on 26 August 2024 [12], ‘*The number of people with mpox due to MPXV clade I has increased in recent months alongside a geographical expansion of mpox in African countries where it was not previously documented. More imported mpox cases due to MPXV clade I are likely to be reported by EU/EEA and other countries*’.

Therefore, laboratory capacities worldwide should be strengthened in preparation for a potential increase of mpox cases caused by MPXV clade Ib infection. It is especially important to be able to reliably diagnose, but also to type MPXV clade Ib, in order to track spread, understand transmission dynamics and implement appropriate containment measures. As highlighted by Schuele et al., existing MPXV real-time PCR-test kits might have reduced sensitivities towards the novel clade Ib because of a deletion in the virus genome. Therefore, thorough validation of commercial and existing in-house kits is warranted to avoid misdiagnosis of cases [13]. Furthermore, PCR-based assays that allow rapid diagnosis and MPXV sub-typing would be advantageous, as at the moment, whole genome sequencing is the method of choice. In addition, it is also crucial to ensure that sufficient quantities of diagnostic kits are available. This is especially important for countries in Africa, as further spread of the outbreak within the continent and outside should be prevented by supporting local African health systems in their efforts to containing the outbreak.

## Conclusion

The EAC mobile laboratory network, fully integrated in NPHL operations, is an important laboratory resource for the EAC under the responsibility of the national public health institutions. This network could also be of interest for international agencies that support outbreak investigations as well as a research infrastructure to conduct studies within an outbreak setting.
